# Recurrent Osteomyelitis in a Paediatric Patient with a Novel *NTRK1* Mutation: A Case Report on Congenital Insensitivity to Pain with Anhidrosis

**DOI:** 10.3390/children12030344

**Published:** 2025-03-09

**Authors:** Liena Gasina, Nityanand Jain, Arturs Viksne, Dzintars Ozols, Mohit Kakar, Uldis Bergmanis

**Affiliations:** 1 Faculty of Medicine, Riga Stradinš University, LV-1007 Riga, Latvia; nityapkl@gmail.com; 2 Department of Paediatric Surgery, Children’s Clinical University Hospital, LV-1004 Riga, Latvia; arturs.viksne@bkus.lv (A.V.); dzintars.ozols@bkus.lv (D.O.); mohits.kakars@bkus.lv (M.K.); 3 Department of Paediatric Surgery, Riga Stradinš University, LV-1007 Riga, Latvia; 4 Department of Orthopaedics & Traumatology, Children’s Clinical University Hospital, LV-1004 Riga, Latvia; uldis.bergmanis@bkus.lv

**Keywords:** congenital insensitivity to pain with anhidrosis, CIPA, hereditary sensory and autonomic neuropathy type IV (HSAN IV), chronic osteomyelitis, case report, complications, paediatric patient

## Abstract

Background: Congenital insensitivity to pain with anhidrosis (CIPA), also known as hereditary sensory and autonomic neuropathy type IV (HSAN IV), is an exceedingly rare genetic disorder characterized by the inability to perceive pain, inability to sweat, and various neurological and orthopaedic complications. Case Presentation: This is a case report of a 3-year-old female patient as the first case in Latvia diagnosed with CIPA syndrome who repeatedly presented to Children’s Clinical University Hospital (CCUH) in Riga, Latvia, with severe orthopaedic manifestations. The patient had repeated fractures, several surgeries, and extensive spread of the disease throughout the left leg, which caused significant functional impairment and decreased quality of life. Despite aggressive orthopaedic interventions, including surgical interventions and physical therapy, the patient’s condition remained challenging to manage due to the inherent limitations posed by the insensitivity to pain. The Surgeon–Radiologist Council of Doctors discussed the patient’s condition and clinical sequalae, deciding that reconstructive surgery is not feasible, and amputation is recommended. Conclusions: Through this case report, we aim to highlight the unique orthopaedic challenges encountered in the management of CIPA patients, emphasizing the importance of a multidisciplinary approach involving orthopaedic surgeons, paediatricians, geneticists, and physiotherapists. Additionally, we discuss the need for further research to elucidate optimal management strategies and improve outcomes in this rare and complex patient population.

## 1. Introduction

Congenital insensitivity to pain with anhidrosis (CIPA), also known as hereditary sensory and autonomic neuropathy type IV (HSAN IV), is an extremely rare autosomal recessive disorder characterized by the inability to perceive pain, absence of sweating (anhidrosis), and various neurological abnormalities [1]. First described in 1963 by Swanson, the syndrome has since been reported in scattered cases worldwide, with the prevalence of disorder in Japan being estimated to be at 1 case in 600,000–950,000 individuals [2]. CIPA patients frequently demonstrate a pattern of recurrent injuries, infections, and self-mutilation, with a notable prevalence during infancy and childhood. Additionally, CIPA patients are susceptible to hyperthermia due to their impaired thermoregulatory mechanisms within the body [3]. It is noteworthy that functions such as salivation, lacrimation, and touch perception remain uncompromised in such patients.

A combination of nerve biopsy, genetic testing, and clinical assessment contributes to the diagnosis. CIPA has been associated with genetic mutations that disrupt the function of nerve fibres responsible for transmitting pain, temperature, and sweat sensations. Therefore, genetic testing of the neurotrophic receptor tyrosine kinase 1 (*NTRK1*) gene is required in most patients to confirm the specific mutation and validate the diagnosis [4]. The *NTRK1* gene encodes the high-affinity nerve growth factor receptor tropomyosin receptor kinase A (TrkA) that plays a crucial role in the development and survival of sensory and sympathetic neurons [5,6]. Beyond its role in neuronal development, *NTRK1* also plays a role in immune regulation, which may contribute to the dysregulated immune response and impaired infection resolution seen in children with CIPA [7,8].

To date, more than five hundred functional mutations, including missense, stop-gain, and frameshift mutations, have been reported in the *NTRK1* gene, of which at least 105 are reported to be associated with CIPA [5]. As demonstrated in a cell-culture based study, every mutation is associated with a distinct TrkA conformation [9]. Each conformation possesses unique characteristics in terms of cellular toxicity profiles, degradation half-lives, and expression in distinct neuronal populations within the brain. These findings potentially explain the varied clinical phenotypes seen in CIPA patients and underscores the importance of genetic testing for diagnosis and patient management [9].

The primary goals of CIPA treatment are symptom management and the early prevention of complications. Interventions are meant to improve the quality of life for those affected and include proactive steps to prevent hyperthermia, supportive therapy, pain avoidance education for the child and the parent, and attentive wound care. Potential therapeutic approaches, such as gene therapy and pharmaceutical therapies that target neurodevelopment pathways, are being investigated in the ongoing research [10].

Herein, we present the first documented case of CIPA from Latvia, a Baltic country located in Eastern Europe. It is noteworthy that our patient presented with an intronic mutation in the *NTRK1* gene that has been documented in only one other patient [11], and a novel missense mutation in the *NTRK1* gene that has not been previously reported in the literature. As such, we believe that our report contributes to the expanding body of knowledge in this field and would provide the healthcare providers in the region with guidance on diagnosing and managing CIPA patients, given the limited understanding of the disease pathogenesis.

## 2. Case Report

A 3-year-old female paediatric patient with known mutations in the *NTRK1* gene (Table 1) was presented to the Regional Hospital in Ventspils, Latvia, in July 2023. According to the patient’s medical history, she was diagnosed with CIPA at the age of two months. Her parents reported that she does not experience pain or sweating. The patient is a child of non-consanguineous parents. The patient has been observed to exhibit recurrent self-injurious behaviour, characterized by the deliberate tearing of the skin from her fingers, and damage to her buccal mucosa and tongue. The patient’s mother had expressed concern, noting that the abrasions tended to not heal adequately; the patient was prone to frequent superficial cutaneous, oral, and genitourinary infections; and that the patient often had an elevated body temperature. There was also a known history of osteomyelitis in the bones of the hand that was treated conservatively using antibiotics.

The patient also had delayed language development. Her vocabulary encompassed both English and Latvian, yet she was not able to form coherent sentences. The parents had reported that the patient learned a new word on a weekly basis, and speech therapy classes were scheduled. A hearing evaluation yielded normal results. The patient was noted to be sensitive to bright light, often requiring sunglasses, but no ophthalmological evaluation had been conducted. About a month prior to admission to the Regional Hospital, the patient sustained a fall in the playground. As the patient did not report any pain or functional limitations at the time of the incident, the parents did not seek medical attention until a few days later, when the patient refused to ambulate. Consequently, a left leg tibial fracture was identified and treated conservatively with circular casting for a period of six weeks in another hospital.

### 2.1. Admission to the Regional Hospital, Latvia

**Figure 1:**
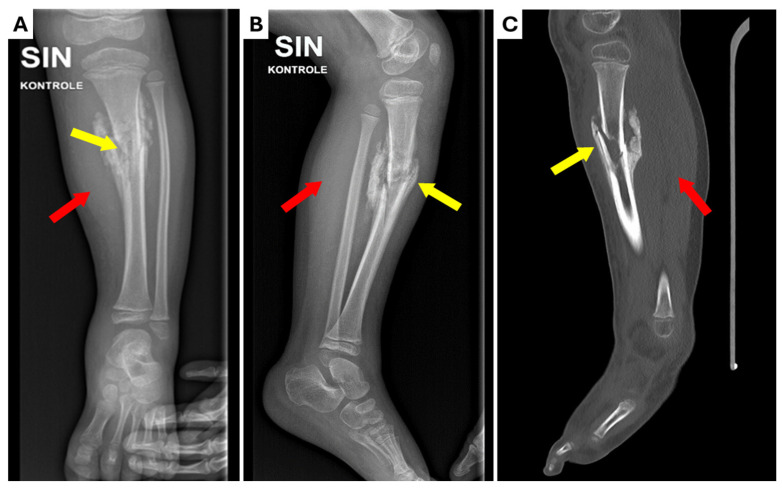
Radiological examinations performed at the Regional Hospital. (**A**) X-ray anterior–posterior projection; (**B**) X-ray lateral projection; (**C**) computed tomography (CT) scan of the lower left leg. Marked sclerotic hyperostosis in soft tissue (red arrows) around the fracture zone and non-union of tibial mid-diaphyseal fracture (yellow arrows) with moderate angular deformation (~15^o^) can be seen.

At the end of the fifth week of immobilization, the patient suddenly developed a persistent fever (temperature T ~40 °C) that did not respond to over-the-counter antipyretics and complained of a sore throat. The parents rushed the child to the Regional Hospital where a control X-ray of the leg was performed after the removal of the cast under sedation. The X-ray showed marked sclerotic hyperostosis in the surrounding soft tissues, non-union of the tibial fracture, along with moderate angular deformation. A subsequent computed tomography (CT) scan of the leg corroborated the findings (Figure 1). Inflammatory markers were found to be elevated (C-reactive protein (CRP): 150 mg/L, procalcitonin: 19 ng/mL). Osteomyelitis with sepsis was suspected and the patient was started on clindamycin (200 mg i/v) and oxacillin (700 mg i/v). Ibuprofen *per os* was prescribed for fever. Simultaneously, blood samples for cultures were collected, and it was decided to transfer the patient to our tertiary hospital for specialized care.

### 2.2. First Hospitalization at the Children’s Clinical University Hospital (CCUH)

On the following day, the patient presented at our hospital. We observed pronounced oedema and erythema in the middle one-third of the anterior surface of the tibia, and the patient complained of localized discomfort. The patient had tachycardia (heart rate (HR): 128 ×/min) and showed symptoms of lethargy, daytime sleepiness, decreased appetite, and minimal fluid intake (<200 mL), although urination was adequate. Patient’s mother complained that the child had constipation since the past couple of days. Capillary refill was normal, and the skin was warm and pale. Lung auscultation revealed that breathing was symmetrical, devoid of any abnormalities, and there were no indications of coughing or nasal congestion. Laboratory investigations continued to indicate elevated inflammatory markers (WBC: 16,000 cells/μL, procalcitonin: 19 ng/mL, and CRP: 150 mg/dl).

**Figure 2:**
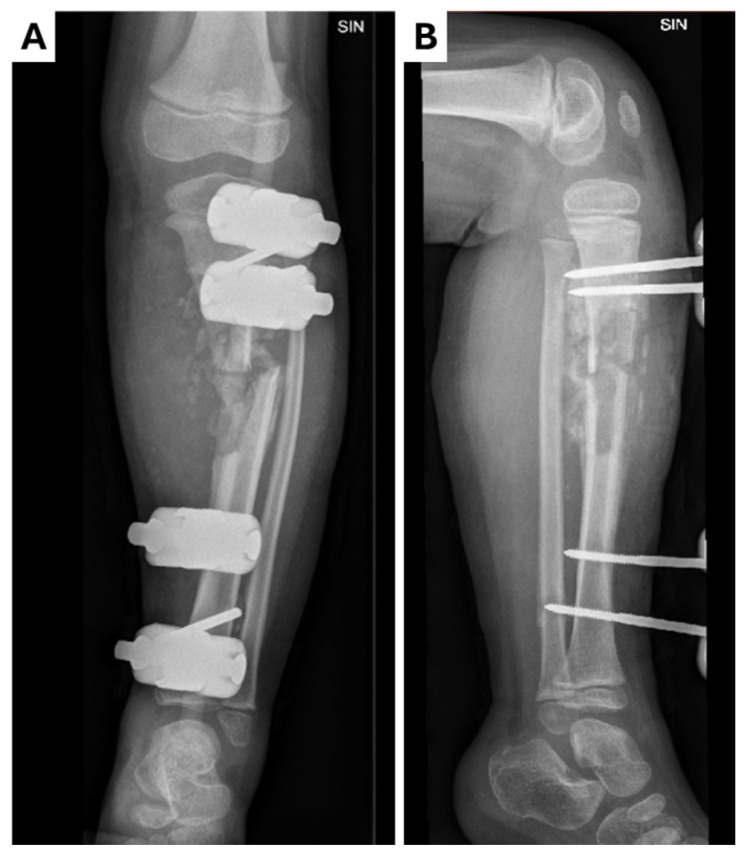
Control X-ray of left leg in (**A**) anterior–posterior and (**B**) lateral projections after surgical manipulation and external fixation. During surgery, the rotation of the medial part of the tibia toward the fracture was performed along with the drainage of pus (~500 mL) around the fracture site. Pseudoarthrosis was observed during surgery along with the presence of infected cartilage tissue. Resection of the damaged bone was performed, resulting in limb shortening (~1 cm). A chlorhexidine impregnated material was left in the wound.

As no improvements in the patient’s dynamics were observed over the next 24 h, a surgical approach was considered: partial resection of the left tibia, cavity repair, and application of an external fixation device (Figure 2). The surgical site tissue and resected bone fragments were sent for histopathologic and microbiologic examinations. The patient was transferred to the Intensive Care Unit (ICU) for further monitoring and was administered metamizolum i/v 250 mg three times a day for pain management. A control echocardiogram (ECHO) performed in the ICU revealed mild mitral regurgitation, patent foramen ovale, and bilateral low-volume hydrothorax. Soft tissue cultures from the surgical site and blood cultures from the Regional Hospital both revealed *Staphylococcus aureus* infection (methicillin-sensitive, penicillin-sensitive). Oxacillin at 1000 mg six times a day was initiated based on the infectologist’s recommendations. The histopathological examination reported a morphological appearance consistent with acute osteomyelitis.

Post-operatively, although the patient was stable (T 37 °C, HR 85 ×/min, SpO_2_ 97%, and normal capillary refill), a lung ultrasound showed fluid in the pleural space (3 cm, non-puncturable region) with radiographic foci of consolidation on the left side consistent with septic pneumonia. The patient was administered fentanyl at 0.5 mcg/kg/hr for pain management and furosemide to induce diuresis. Because purulent contents were observed in the surgical cavity, the patient was placed on a vacuum-assisted closure (VAC) device, followed by regular wound care. She remained in the ICU for one week before being transferred to the surgical ward. A microbiological examination of the pus and wound surgical material revealed *S. aureus* infection (antibiogram is presented in Table 2). Throughout this period, the patient episodically refused to eat, became restless and agitated, refused to cooperate with nurses, and banged both legs against the bed. A nasogastric (NG) tube was placed, and mild sedatives were prescribed as needed.

One week later in the surgical ward, her NG tube was evacuated and the patient started eating and drinking *per os* on her own. The lower-extremity wound had developed signs of granulation tissue, and a closure procedure was performed by rotation of the gastrocnemius and soleus musculocutaneous flaps. No significant deterioration of the patient’s dynamics was observed during the following weeks of observation. In a compensated satisfactory condition, the patient was discharged for outpatient therapy with a diagnosis of hematogenous osteomyelitis of the left proximal tibia.

### 2.3. Second Hospitalization at the Children’s Clinical University Hospital (CCUH)

**Figure 3:**
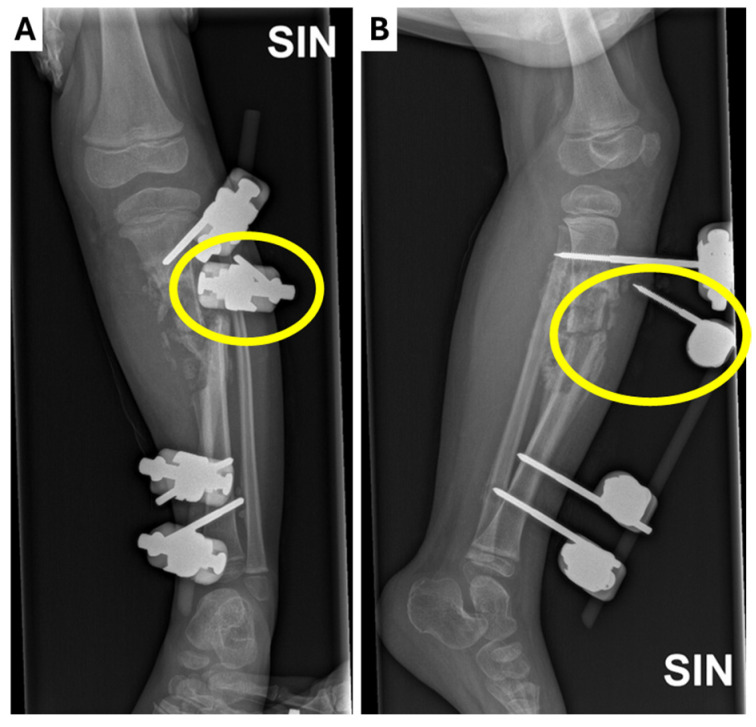
Radiological examinations of the left leg showing the dislocation of one of the external fixators (yellow circle) in the (**A**) X-ray anterior–posterior projection; (**B**) X-ray lateral projection.

One week after discharge, the patient was playing on the couch at home when her leg got stuck, causing instability and the dislocation of the external fixator (Figure 3). She was admitted acutely with purulent discharge from the wounds. We evacuated the rods of the external fixator from the bone and sent the biological material for microbiological examination. Blood cultures were negative for microflora and no signs of active inflammation were noticed in the laboratory tests. The wound was cleaned and immobilized with a plaster cast. Prophylactic clindamycin at 200 mg three times a day was prescribed along with ibuprofen *per os*. The patient was discharged on the third post-operative day. A planned follow-up in three weeks was recommended.

### 2.4. Third Hospitalization at the Children’s Clinical University Hospital (CCUH)

**Figure 4:**
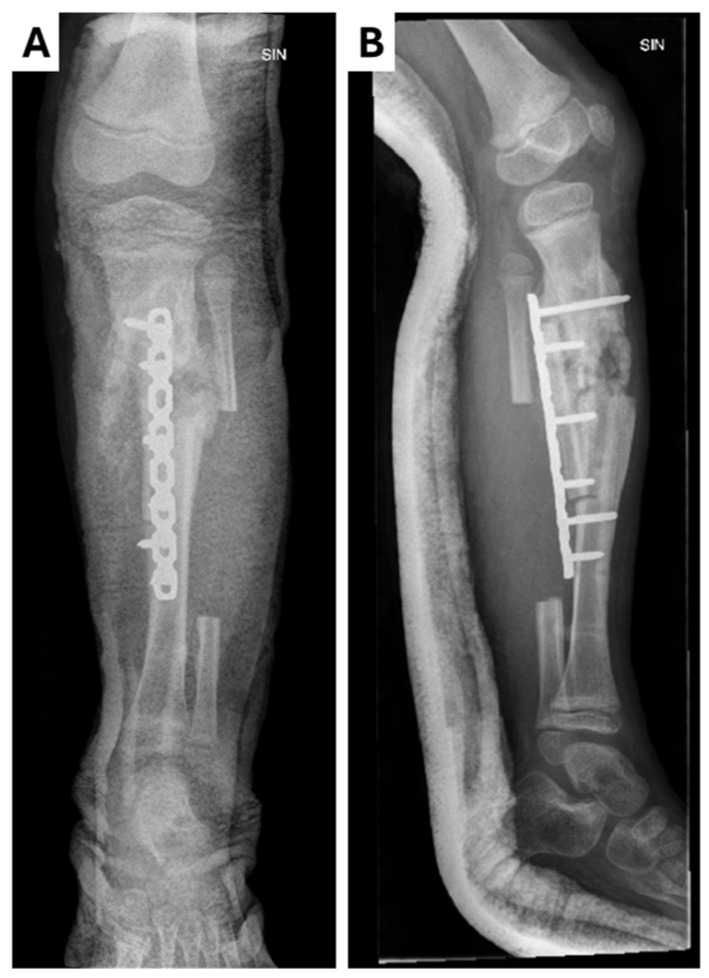
Radiological examinations of the left leg showing chronic osteomyletic changes in the tibial diaphysis in the (**A**) X-ray anterior–posterior projection; (**B**) X-ray lateral projection. A proximal–distal osteotomy of the tibia was performed, resecting the pseudoarthrosis and exposing the bone surfaces. A proximal–distal osteotomy of the fibula was also performed, along with the rotation of the bone fragments to place them in the defect around the pseudarthrosis. Osteosynthesis was performed with a locked plate and five screws while maintaining the axis of the bone. A sterile aseptic dressing and plaster cast were applied.

The patient underwent scheduled admission one month later for follow-up lower leg reconstruction: osteotomy, osteonecretomy, pseudoarthrosis plasty with a vascularized fibular graft, and osteosynthesis with a locking plate (Figure 4). The patient was prescribed clindamycin. The next day, the patient developed a fever (T 40 °C) with swelling, redness, and pain in the leg (CRP 119 mg/L). An acute surgical revision was performed. The wound was clean, and no purulent discharge, no hematoma, and no significant signs of infection were observed. Cefuroxime was added and the patient was admitted to the ICU. However, the fever did not subside over the next 24 h, so the antibiotic was changed to piperacillin/tazobactam. The fever gradually resolved over the next two days and the patient was transferred back to the ward. A decompression incision was made later in the day. There was no discharge from the wound and no signs of infection. The removed cast was reapplied to the leg. A week later, the wound site was surgically closed and monitored for one week before discharge. Instructions were given to continue cast immobilization for an additional eight weeks.

### 2.5. Fourth Hospitalization at the Children’s Clinical University Hospital (CCUH)

Two months later, the patient was admitted to another hospital because of the swelling of the distal part of the left leg and the area around the left ankle. She was diagnosed with fractures of the lateral and medial malleolus of the left leg and started on rifampicin and cefazolin. She also underwent aspiration of the left knee, and a VAC device was placed due to localized fluid accumulation. During her three-week hospitalization, radiologic investigations revealed a new focus of osteomyelitis in the distal part of the left femur, and she was transferred to our hospital. Upon arrival, the VAC device was evacuated, and serous discharge was observed. Discharge cultures were positive for *Staphylococcus epidermidis* infection. Antibacterial therapy was started accordingly (Table 2).

**Figure 5:**
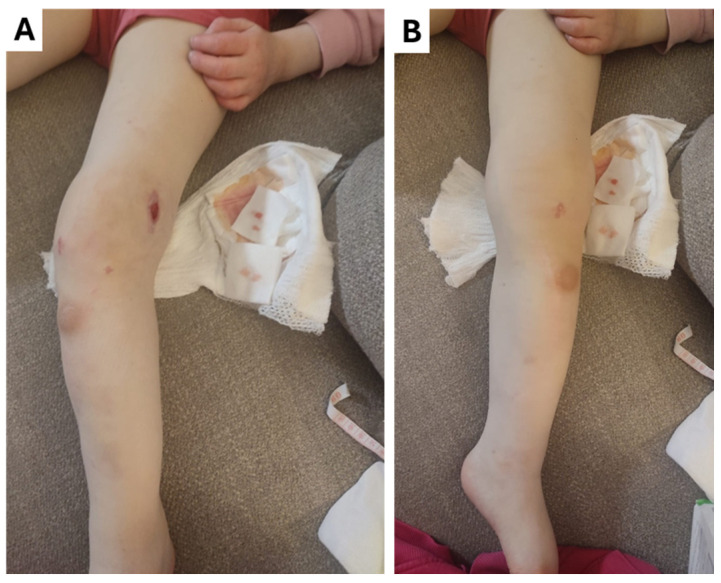
(**A,B**) The appearance of the patient’s left leg four weeks after discharge from the hospital showing pronounced left knee valgus, flexion of the hip joints, and a localized wound on the lateral surface of the left thigh.

Given the nature and extent of the radiographic changes, the complexity and duration of the condition, the patient’s condition was discussed in the Surgeon–Radiologist Council of Doctors, where it was decided that further reconstructive surgery was not practical, and the parents were advised to consider the possibility of amputation. The parents declined amputation, and the patient was discharged with recommendations for continued antibacterial therapy. At the one-month follow-up, the patient had left knee valgus and hip flexion (Figure 5). After a consultation with a physiotherapist and an orthopaedist, it was determined that the passive range of motion of the ankle joint was normal and the range of motion of the foot and the knee joint was increased. Pronounced valgization of the knee during gait caused recurrent inflammation and the patient was recommended to use hard knee–ankle–foot orthosis.

### 2.6. Fifth Hospitalization at the Children’s Clinical University Hospital (CCUH)

**Figure 6:**
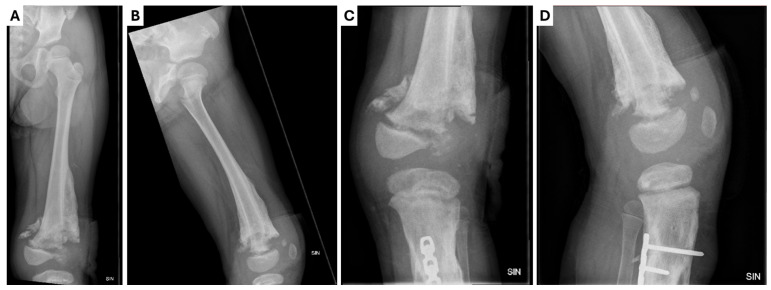
X-ray examination of the (**A,B**) left hip joint and (**C,D**) femur and knee joint in anterior–posterior and lateral projections showing a small, heterogeneous lateral condyle of the femur, a lytic zone in the metaphysis, massive calcification in the mid-diaphysis, a normal hip joint gap, smooth surfaces, and no pathologies in the hip joint bones.

Approximately 3 months after the last hospitalization, the patient presented again as an acute patient with complaints of subfebrile body temperature and discomfort in the left hip joint, refusing to lean on the left leg. Inflammatory markers were elevated (CRP: 106 mg/dl). The localized wound on the lateral surface of the left thigh did not heal and wound and blood samples were taken for culture. Both were negative for microflora. An X-ray examination was conducted on the patient’s left hip joint, femur, and knee joint (Figure 6). Antibacterial therapy with vancomycin at 300 mg i/v three times a day, cefotaxime at 750 mg three times a day, and oxacillin at 550 mg four times a day was initiated. The patient’s condition gradually improved after six days of antibacterial therapy, and no new foci of osteomyelitis were found. The patient resumed leaning on her left leg and was discharged from the hospital with recommendations to continue using a rigid orthosis and to continue regular bandaging of the wound.

## 3. Discussion

The present case report presents our experience with a young female patient diagnosed with CIPA and underscores the necessity of a multidisciplinary approach for the diagnosis and management of such patients. The absence of pain and typical inflammatory signs complicates early interventions. Given the rarity and complexity of CIPA, the involvement of a specialized team comprising orthopaedic surgeons, paediatricians, pain specialists, psychologists, and rehabilitation experts is necessary to ensure the development of tailored, personalized, and optimized diagnostic strategies and treatment plans [12,13]. This is particularly important for CIPA patients, given the wide range of clinical presentations reported in the literature [14,15]. We believe that our report provides valuable insights into CIPA’s clinical features and management strategies. It underscores the importance of a multidisciplinary approach and serves as an educational resource for healthcare professionals.

### 3.1. Diagnostic and Therapeutic Patterns in CIPA Patients

Pain insensitivity is known to significantly delay injury recognition in CIPA patients, as evidenced by our case where a tibial fracture progressed to osteomyelitis without associated physical symptoms, such as redness and pain. The frequent occurrence of self-inflicted musculoskeletal injuries, including unnoticed fractures, exacerbated pressure ulcers, and infections that could lead to sepsis, underscores the necessity for vigilant monitoring in such patients. In our case, the patient’s reluctance to walk and emotive agitation alerted the caregivers to the injury, despite the patient’s reported lack of pain.

Physical signs, such as swelling and restricted movement, further aided in identifying injuries, highlighting the importance of a comprehensive clinical examination. Radiological imaging was also found to be crucial for detecting osteolytic changes, while general inflammatory markers, such as CRP and the erythrocyte sedimentation rate (ESR), were sometimes unreliable [16]. Nabiyev et al., documented multiple cases of osteomyelitis in CIPA patients, emphasizing the challenges of early diagnosis and the necessity for prolonged treatment due to delayed detection [17]. Zhang and Haga reported that 65% of CIPA patients experienced skeletal complications, with 91% of all reported fractures affecting the lower limbs, commonly seen in young children between the ages of one and seven years [18].

Decisions guiding orthopaedic interventions require a balanced consideration between conservative and surgical approaches. As reported by Hartono et al., articular and metaphyseal fractures in CIPA patients treated surgically often result in complications, such as infections and non-union. In contrast, diaphyseal fractures have shown better healing outcomes with conservative management, such as cast immobilization, highlighting the importance of careful treatment selection in CIPA patients [19]. Nonetheless, the initial conservative treatment of fractures with casts may prove to be effective only in mild cases [20] due to poor patient compliance, recurrent localized infections, and delayed wound healing. External fixation often also proves to be ineffective due to mechanical instability, necessitating multiple surgical revisions [21].

During the five hospitalizations that spanned a period of nine months, our patient developed valgus deformity and hip flexion contracture, both of which resulted in significant impairments to the patient’s mobility. This deterioration in her physical capacity was further compounded by the presence of gait abnormalities, which required rigid orthotic support. The rapid progression of complications shows the importance of parental education and vigilant monitoring in facilitating the implementation of early intervention strategies. Prolonged antibiotic therapy, often guided by culture results, is crucial for managing recurrent infections and preventing sepsis. In agreement with a previous report from a retrospective cohort study that identified *S. aureus* as the most prevalent pathogen isolated in CIPA patients, our patient also exhibited recurrent *S. aureus* infection. However, in contrast to 48.5% of cases reported as methicillin-resistant in their study, our patient’s strain was methicillin-sensitive [22].

### 3.2. Complementary Management Strategies

Managing CIPA presents significant challenges due to the absence of pain perception, delayed injury recognition, and a high risk of recurrent infections and fractures. Fractures in children with CIPA often heal poorly, forming hypertrophic pseudoarthrosis. A fracture that typically heals in three weeks may take six months or fail to heal in these patients. They are also prone to bone and soft tissue-resistant infections [23]. Given these challenges, hyperbaric oxygen therapy has been suggested as a potential adjunct to promote bone healing and infection control [24]. Bisphosphonate therapy has also been used in CIPA patients with recurrent fractures, showing promising results in fracture prevention. For example, a case report described intravenous pamidronate treatment over one year, which successfully reduced fractures in the upper and lower limbs, skull, and spine with no observed adverse effects at five years of follow-up [25].

Experimental therapies, such as gene therapy, nerve growth factor administration, or stem cell-based approaches, may offer future solutions to enhance neuronal function and reduce complications in CIPA patients [10]. Based on our experience and reports in the literature, complementary strategies can improve outcomes and reduce long-term morbidity in CIPA patients, including:

- *Enhanced infection control* using early and targeted antibiotic therapy guided by culture and sensitivity testing. Long-term suppressive antibiotic therapy and aggressive wound management, such as vacuum-assisted closure (VAC) therapy, may mitigate infection risks.
- *Bone grafting*, including vascularized fibular grafts or synthetic bone substitutes, to promote structural stability and reduce non-union risks.
- *Custom orthotic solutions*, such as advanced orthoses, tailored to the patient’s biomechanical needs that can improve mobility and reduce pressure ulcer and deformity risks.
- *Early amputation* remains controversial in the literature. Amputation decisions in CIPA patients involve a careful consideration of the patient’s and family’s preferences. Discussions should address the rationale, alternatives, and expected outcomes, providing support throughout. Risk–benefit analysis is essential, weighing benefits, such as mobility against surgical and psychological risks. Amputation may be warranted when limb salvage leads to recurrent hospitalizations and a poor quality of life. The literature indicates that while parents often resist amputation, it may become necessary as the disease progresses [26,27]. Additionally, a high risk of auto-amputation due to self-harm has been reported by others [28,29,30].
- *Multidisciplinary rehabilitation* that acts as a structured rehabilitation program, including physiotherapy, occupational therapy, and psychological support. It is crucial for improving functional outcomes and adaptation. Repeated surgical interventions in paediatric patients, particularly those with chronic conditions like CIPA, not only pose significant physical challenges, but also contribute to psychological distress. Studies have shown that up to 16% of children and 23% of parents develop post-traumatic stress disorder (PTSD) following paediatric surgeries. The increased risk is linked to prolonged hospital stays, multiple procedures, and insufficient psychosocial support [31].

### 3.3. Prognosis and Follow-Up Care

Given the progressive and chronic nature of CIPA, lifelong follow-up is essential for monitoring skeletal deformities, infections, and neurological complications. CIPA patients can survive into adulthood, with reported ages ranging from 6 months to 33 years. However, mortality remains a concern, with fatalities often resulting from septic shock or post-surgical cardiac arrest linked to dysautonomia [22]. Regular radiographic assessments, infection monitoring, and orthopaedic evaluations should be integral to follow-up care. This is illustrated by a case report that documented a 7-year follow-up for a patient with CIPA, resulting in the development of progressive acro-osteolysis [32]. This suggests that patients with CIPA syndrome should be under the supervision of specialists for the duration of their lives, as the disease is progressive in nature. The use of custom orthotic solutions and adaptive physical therapy can improve long-term functionality and mobility. Psychological support is also essential to address behavioural issues related to self-harm and sensory-seeking behaviours [32].

Our search of the literature led us to identify one of the mutations being previously reported in a 7-year-old Turkish male child [11]. The child was born to non-consanguineous parents who were heterozygous mutation carriers. Comparing the clinical sequelae of that patient to ours, we observed that both patients had complained of frequent episodes of fevers, delayed speech, eczematous lesions, hyperkeratosis, and signs of self-mutilation in and around the oral cavity. Furthermore, both children had reported multiple tibial and ankle fractures at a young age. However, the authors did not elaborate on how these fractures were managed and/or what complications were observed [11], highlighting the lack of literature surrounding the proper management of CIPA patients.

Additionally, unlike the Turkish patient, our patient did not show signs of ataxic gait and convulsions, highlighting the possibility of observing them as the patient ages. In our case, the patient’s history of osteomyelitis, fractures, and joint instability also increases her risk of limb deformities, mobility loss, and chronic infections in the future. Progressive valgus knee deformity and hip flexion contracture may lead to wheelchair dependence. Recurrent *S. aureus* infections heighten the risk of sepsis. Additionally, her anhidrosis makes her susceptible to life-threatening hyperthermia. While amputation has been initially refused by the parents, it remains a viable option to prevent further deterioration in the future.

### 3.4. Future Directions

The research on CIPA remains limited, with most of the literature comprising case reports focused on new cases and patient phenotypes. This highlights the need for further studies exploring treatment strategies and long-term outcomes [33]. Furthermore, there is a need to summarize the findings and management tactics from reported case studies to understand CIPA’s full clinical and genetic spectra. Long-term follow-up studies are also needed to elucidate patient outcomes and risk factors for disease progression. Future research should also explore early diagnostic markers, optimized therapeutic strategies, and standardized treatment protocols to improve long-term outcomes.

Investigations into bone regeneration, nerve growth factor therapies, and advanced orthopaedic interventions, such as implants, may offer new possibilities for managing CIPA-related complications. Alternative pain management approaches, including non-pharmacological interventions, should be explored to improve quality of life. Enhanced genetic counselling and early screening can aid in monitoring and managing complications. Collaboration among researchers, clinicians, and patient advocacy groups is essential to advance knowledge. Multicentre and international collaborations can accelerate the research and develop consensus guidelines for managing CIPA. Ethical and sociolegal dilemmas in invasive interventions, like amputation, should continue to be explored, and psychosocial support should be integrated into care plans to address the emotional and social impacts of living with a rare genetic disorder.

## 4. Conclusions

Early diagnosis of CIPA syndrome is crucial to prevent complications, as personalized treatments are not available and surgical interventions are often challenging, not always achieving the desired results. Chronic osteomyelitis is a severe orthopaedic complication that requires a robust, multidisciplinary approach. Future research should aim to develop early diagnostic markers and explore new treatments to enhance outcomes and quality of life for CIPA patients.

## Figures and Tables

**Table 1:** Genetic variants for the *NTRK1* gene identified in the patient.

Genetic Variant ^a,b^	Mutation	Zygosity	Parental Origin	Pathogenicity	Previously Reported
NM_001012331.2:c.2188-11G>A; p.?	Intronic	Heterozygous	Maternal	Likely pathogenic	[11]
NM_001012331.2:c.2109C>A; p.Asp703Glu	Missense	Heterozygous	Paternal	Likely pathogenic	-

^a^ Based on the results of the genetic testing, the child was diagnosed with congenital insensitivity to pain with anhidrosis (CIPA; ICD-10 Code: G60.8; ORPHA:642; OMIM:256800). ^b^ Abbreviations: G—guanine; A—adenine; p.?—unknown effect of the mutation at protein level; C—cytosine; Asp—aspartic acid; Glu—glutamic acid.

**Table 2:** Antibiogram for isolated pathogens from patient material during different hospitalizations.

Antibiotic	*S. aureus* *	*S. epidermidis* **
Penicillin	Resistant	Resistant
Erythromycin	Susceptible	Susceptible
Clindamycin	Susceptible	Resistant
Ciprofloxacin	Susceptible	Susceptible at increased exposure
Tetracycline	Susceptible	Susceptible
Gentamicin	Susceptible	Susceptible
Trimetroprim/Sulfamethoxazole	Susceptible	Susceptible
Methicillin-sensitive	Yes	Not applicable
Levofloxacin	Not tested	Susceptible at increased exposure
Amikacin	Not tested	Susceptible
Imipenem	Not tested	Resistant

* Isolated during the first hospitalization from the pus and wound material. ** Isolated during the fourth hospitalization from the wound material.

## Data Availability

All relevant clinical and medical data has been described and presented in the present case report. Further information on the management and follow-up of the patient can be addressed to the corresponding author for non-commercial educational purposes only. Approval and consent of the patient’s parents and other relevant institutions, in accordance with local regulations, would be required before sharing of personal patient data.
